# Pain, fatigue and functional disability are associated with higher educational needs in systemic sclerosis: a cross-sectional study

**DOI:** 10.1007/s00296-018-3998-0

**Published:** 2018-03-01

**Authors:** Matylda Sierakowska, Stanisław Sierakowski, Justyna Sierakowska, Elżbieta Krajewska-Kułak, Mwidimi Ndosi

**Affiliations:** 1 Department of Integrated Care Medical, Medical University of Bialystok, 7a Maria Sklodowska-Curie Street, 15-096 Bialystok, Poland; 2 Department of Rheumatology and Internal Diseases, Medical University of Bialystok, Bialystok, Poland; 3 Department of Foreign Languages, Medical University of Bialystok, Bialystok, Poland; 4 Department of Nursing and Midwifery, University of the West of England, Bristol, UK; 5 Academic Rheumatology Unit, University Hospitals Bristol, Bristol, UK

**Keywords:** Systemic sclerosis, Educational needs, Disability, Pain, Fatigue, Raynaud’s phenomenon

## Abstract

In the process of the planned and systematic education of patients/families, it is extremely important to identify patients’ health problems as well as their needs and expectations. The objective of this study was to determine the relationship between functional disability, health problems and perceived educational needs in people with systemic sclerosis (SSc). This was a cross-sectional analytic study conducted in six rheumatology centers in Poland. Functional disability was measured using HAQ-DI, and the magnitude of other health problems (pain, fatigue, intestinal problems, breathing problems, Raynaud’s phenomenon, finger ulcerations) was measured using 0–100 mm visual analogue scales. The educational needs were measured using the Polish version of the Educational Needs Assessment Tool (Pol-ENAT). Spearman’s correlation coefficient (*r*_s_) was used to report associations. The sample comprised 140 patients, 125 (89.28%) were women. They had a mean (SD) age of 54 (14.23) and disease duration of 11 (10.27) years. The median (IQR) HAQ-DI was 1.12 (0.62–1.62) and mean ENAT score was 71.54 (SD 27.72). Patients needed to know more about the disease process, self-help measures and managing pain. All health problems had significant correlations with the overall educational needs, pain, functional disability and fatigue having the highest *r*_s_ = 0.359, *p* < 0.0001; *r*_s_ = 0.314, *p* < 0.001 and *r*_s_ = 0.270, *p* = 0.001, respectively. Health problems in people with SSc are associated with considerable educational needs; therefore, health professionals should take this into account when planning patient education. Group interventions should consider providing patient education related to disease process as a minimum.

## Introduction

Systemic sclerosis (SSc), scleroderma, is a complex heterogeneous chronic condition with an autoimmune background, characterized by progressive fibrosis of the skin and internal organs, which lead to their failure, as well as abnormal renal morphology and dysfunction of blood vessels [1]. Scleroderma is considered a rare disease affecting between 31 and 88 per 1,000,000 population in the UK [2] and around 250 per 1,000,000 in Poland [3]. Women are 3–5 times more likely to be affected than men.

Several clinical subsets exist and differ by skin involvement, frequency and the extent of organ involvement, the presence of antinuclear antibodies, the nature of microvascular changes as well as the disease’s course and prognosis. The most common is limited systemic sclerosis (approximately 2/3 of patients) and the other subset, diffuse systemic sclerosis is more severe and affects approximately 1/3 of all patients with SSc. Due to its multisystem involvement, the disease has severe physical and psychosocial impact affecting the patients’ quality of life [4].

Management of SSc includes patient education aimed at preparing patients and their carers or family for self-management. Given the complexity of the disease, potential complications and treatments [5], self-management of the SSc will involve more than just self-care. This is likely to include focusing on the disease and its needs, knowledge and engagement with healthcare and managing life with the chronic illness [6]. Needs-based patient education is the means through which effective self-management could be achieved [7].

Patient education is an integral component of the comprehensive management of rheumatic diseases and this is consistent with the recommendations of the European League Against Rheumatism [7] and the diagnostic and therapeutic standards of the Polish Society for Rheumatology [8]. Patient education can be defined as a planned interactive learning process designed to support and enable people to manage their life with a rheumatic disease and optimise their health and well-being [7]. It comprises all educational activities provided for patients, including aspects of therapeutic education, health education and health promotion [9]. Its benefits include improved knowledge about the disease [9], increased self-efficacy [10], acceptance and motivation to take actions that positively influence the health of the patient.

It is important to identify the needs and expectations of the patient, as well as their health problems and difficulties, especially in their physical, psychological, and social functioning. The success of the complex therapeutic treatment requires that the patient with SSc is actively engaged in the process of treatment and in the monitoring of the pharmacological therapy’s effects [5, 8]. The patient should know the drugs’ adverse effects as well as be able to observe the internal organs’ symptoms and any signs of exacerbations. It is of utter importance to prevent the complications and life-threatening states, such as scleroderma renal crisis or pulmonary hypertension.

The interdisciplinary rheumatology team needs to ensure that targeted, needs-based patient education is given to all patients [7]. The educational needs assessment tool (ENAT) was developed to enable systematic assessment of educational needs of people with rheumatic disease [11]. This tool has now been validated in Poland for use in SSc [12]. The aim of this study was to determine the relationship between functional disability, health problems and perceived educational needs of people with SSc. This would help understand the factors associated with patient education and determine possible areas for targeting patient education, thus optimize its effects. Targeted education can bring about better functioning, coping and improved quality of life of people with SSc.

## Methods

### Design

This was a cross-sectional analytic study conducted at rheumatology and internal medicine departments of six hospitals in Poland. Ethical approvals were obtained from the Bioethics Committee of the Medical University of Bialystok (R I-002/87/2015).

### Patients and measures

Patients were selected using random sampling and the inclusion criteria were a diagnosis of SSc, based on the ACR/EULAR 2013 criteria [1], and age ≥ 18 years. Exclusion criteria were minimal, only age below 18 years. The patients signed informed consent to participate in the study. Researchers from the centers explained the purpose of the study and the patients completed the questionnaires unaided.

We used the following measures: (i) The Polish version of the educational needs assessment tool (Pol-ENAT) [12] to assess educational needs (ii) health assessment questionnaire disability index (HAQ-DI) (website: http://www.mapi-research-inst.com) [13, 14] to assess functional disability and (iii) 0–100 mm visual analogue scales (VAS) to assess pain severity, fatigue severity and severity of the following health problems: disease severity, intestinal problems, breathing problems, Raynaud’s phenomenon and fingers’ ulceration.

The ENAT is a questionnaire designed to help clinicians identify priority educational needs and help addressing them in a timely manner [11, 15]. The ENAT was first developed for patients with arthritis [15] and was later validated in other rheumatic diseases including SSc [11]. The cross-cultural validation into Polish is reported elsewhere [12]. The tool comprises 29 items, which are formatted as a 0–4 rating scales (0 = not at all important, 1 = a little important, 2 = fairly important, 3 = very important, 4 = extremely important). The items are then grouped into seven sub-scales: managing pain (0–24), movement (0–20), feelings (0–16), disease process (0–28), treatments (0–28), self-care measures (0–24) and support systems (0–16). The ENAT domain scores are Rasch-transformed (using raw-to-linear score conversion charts—published elsewhere [12]) to enable their use in parametric analyses. Adding up all the transformed domain scores gives the total ENAT score, which is an estimate of the patient’s educational, needs (range = 0–156) [11, 12]. The HAQ-DI is a validated generic measure of physical functioning combining eight domains (dressing and grooming, arising, eating, walking, hygiene, reach, grip and other activities) [13]. Responses to each item range from zero (no difficulty) to three (unable to do). The total score ranges from 0 to 3. Data collection were carried out from June 2014 to December 2014.

### Data analysis

All data were analysed using PQStat v.1.4.2 software. The raw scores from the Pol-ENAT which are ordinal in nature were transformed into interval level measures using calibrated conversion tables (published elsewhere [12]) before being combined with other data and subjected to parametric analyses. All data were then summarised descriptively using means, standard deviations (interval level data) and medians and interquartile ranges (ordinal-level data) as appropriate. As each ENAT domain has different score range, each ENAT domain scores was “normalized” by dividing by the maximum possible score (%) to enable comparisons across domains. For the inferential statistics, we tested the null hypothesis of no correlation between educational needs and patient health problems (including disability). Spearman’s correlation coefficient *r*_s_ is reported together with *p*-values, where *r*_s_ of 0–0.19, 0.2–0.39, 0.40–0.59, 0.6–0.79 and 0.8–1 represented very weak, weak, moderate, strong and very strong correlations, respectively. HAQ-DI and VAS scores are presented as median with interquartile range. The association between personal characteristics (gender, age, disease duration), and educational background were tested across all measures. Kruskal–Wallis (H) test was used to assess differences and across patient subgroups.

## Results

The sample comprised 140 patients (Bialystok 60, Lublin 20, Warsaw 19, Poznan 17, Sopot 13 and Srem 11) the majority (125, 89.28%) of whom were women and their mean age was 54.14 (SD 14.23) years. Those with limited SSc were 83 (59.28%) and diffuse SSc 57 (40.72%).

Table 1 presents patients’ characteristics, health problems and educational needs. The educational needs are summarized as ENAT domain scores and the degree of need is expressed as average score divided by the maximum possible score for that domain. Of all patient-reported health problems (0–100 mm VAS), fatigue, Raynaud’s phenomenon and disease severity scored the highest. Most patients had ‘moderate to severe’ disability. The median HAQ-DI score was 1.12 (IQR: 0.62–1.62). Most of the respondents (*n* = 120, 85.71%) wanted to get information to help them manage their SSc and 60.71% (*n* = 85) wanted to know ‘everything’. The mean ENAT score was 71.54 (SD 27.72). The highest educational needs were related to the following domains: disease process, self-help and managing pain.

**Table 1:** Patient characteristics, health problems and educational needs

Variables studied (score range)	Mean (SD) except where stated	Degree of need^a^	Median (IQR: Q25–Q75)
Age	54.14 (14.23)		56 (46–64.25)
Disease duration	10.89 (10.27)		8 (3–15)
Gender—number of women (%)	125 (89.28)		
Educational background			
Basic—number (%)	16 (11.43)		
Secondary—number (%)	56 (40.00)		
Professional—number (%)	32 (22.86)		
Higher education—number (%)	36 (25.71)		
HAQ-DI (0–3)			1.12 (0.62–1.62)
Pain-VAS (0–100)			37.5 (15–61.25)
Fatigue-VAS (0–100)			50 (25–70)
Disease severity (0–100)			50 (25–65)
Raynaud’s attacks (0–100)			50 (10–70)
Intestinal problems (0–100)			7.5 (0–45)
Breathing problems (0–100)			20 (0–50)
Finger ulceration (0–100)			0 (0–30)
Educational needs (Pol-ENAT)			
Managing pain (0–24)	12.40 (5.61)	0.52	
Movement (0–20)	8.88 (5.13)	0.44	
Feelings (0–16)	8.05 (4.41)	0.50	
Disease process (0–28)	15.02 (5.79)	0.54	
Treatments (0–28)	7.73 (5.62)	0.25	
Self-help (0–24)	12.51 (6.47)	0.52	
Support (0–16)	6.95 (3.54)	0.43	
Total ENAT (0–156)	71.54 (27.72)		

*IQR* interquartile range, *Q25* first quartile (25th percentile), *Q75* third quartile (75th percentile)

^a^ENAT domains have different number of items, therefore, the degree of need is determined by average score divided by the maximum possible score for that domain

Table 2 summarises the correlations between health problems (including functional disability, pain and fatigue) and educational needs (ENAT domain scores and total score). Pain and functional disability had the largest degree of association with patients’ educational needs, especially in the ‘movements’ domain; *r*_s_ = 0.462 and 0.408 for functional disability and pain, respectively. All health problems had significant correlations with patients’ overall educational needs. There were ‘weak correlations’ between educational needs and health problems except for breathing problems, which had ‘very weak correlation’.

**Table 2:** Correlation between health problems and specific domains of educational needs

Health problems (score range)	Pol-ENAT domains							Total
	Managing pain	Movement	Feelings	Disease process	Treatments	Self-help measures	Support systems	Pol-ENAT
	*r*_s_ (*p*-value)	*r*_s_ (*p*-value)	*r*_s_ (*p*-value)	*r*_s_ (*p*-value)	r_s_(p-value)	*r*_s_ (*p*-value)	*r*_s_ (*p*-value)	*r*_s_ (*p*-value)
HAQ DI (0–3)	**0.166 (0.050)**	**0.462 (**< **0.0001)**	0.140 (0.100)	**0.254 (0.002)**	**0.270 (0.001)**	0.108 (0.203)	0.137 (0.108)	**0.314 (**< **0.001)**
Pain-VAS (0–100)	**0.307 (**< **0.001)**	**0.408 (**< **0.0001)**	**0.178 (0.036)**	**0.297 (**< **0.001)**	**0.225 (0.008)**	**0.171 (0.043)**	**0.198 (0.019)**	**0.359 (**< **0.0001)**
Fatigue-VAS (0–100)	**0.215 (0.011)**	**0.249 (0.003)**	0.160 (0.060)	**0.186 (0.028)**	**0.227 (0.007)**	0.117 (0.170)	**0.178 (0.036)**	**0.270 (0.001)**
Disease Severity (0–100)	0.144 (0.090**)**	**0.352 (**< **0.0001)**	**0.168 (0.047)**	**0.190 (0.024)**	**0.229 (0.006)**	0.076 (0.371)	**0.186 (0.027)**	**0.272 (0.001)**
Raynaud’s attacks (0–100)	0.152 (0.073)	**0.277 (**< **0.001)**	**0.292(**< **0.001)**	0.163 (0.055)	0.102 (0.231)	0.126 (0.138)	**0.171 (0.044)**	**0.236 (0.005)**
Intestinal problems (0–100)	**0.233 (0.006)**	**0.213 (0.012)**	**0.232 (0.006)**	0.153 (0.071)	0.097 (0.253)	0.163 (0.055)	0.036 (0.672)	**0.215 (0.011)**
Breathing problems (0–100)	**0.188 (0.026)**	**0.192 (0.023)**	**0.239 (0.004)**	0.149 (0.080)	0.140 (0.098)	0.055 (0.522)	0.043 (0.616)	**0.197 (0.020)**
Finger ulceration (0–100)	0.085 (0.319)	**0.277 (**< **0.001)**	**0.250 (0.003)**	0.116 (0.172)	0.160 (0.059)	0.097 (0.254)	**0.242 (0.004)**	**0.237 (0.005)**

Bold values indicate the level of significance *p* ≤ 0.05

*r*_*s*_ Spearman’s correlation coefficient where values of 0–0.19 represent very weak, 0.2–0.39 weak, 0.40–0.59 moderate, 0.6–0.79 strong and 0.8–1.0 very strong correlations

Table 3 summarises the correlations between personal characteristics (age, gender and disease duration) and health problems. Age and disease duration were largely correlated with patient-reported health problems. The exceptions to this were finger ulceration and Raynaud’s attacks. Disease duration was associated with disability, pain, disease severity, intestinal and breathing problems; the largest association being due to the difference between 0 and 5 years and > 20 years groups especially on disability (*r*_s_ = 0.312, *p* < 0.001) and pain (*r*_s_ = 0.308, *P* < 0.001). See Table 3.

**Table 3:** The association between patients’ characteristics (age, disease duration and gender) and their health problems

Variables (score range)	Age groups in years				Effect size *r*_s_ (*p*-value)	Disease duration in years					Effect size *r*_s_ (*p*-value)	Gender		
	≤ 40, *n* = 25 Median (IQR)	41–60, *n* = 64 Meidan (IQR)	> 60, *n* = 51 Median (IQR)	H-statistic (*p*-value)		0–5 *n* = 54 Median (IQR)	6–10 *n* = 38 Median (IQR)	11–20 *n* = 29 Median (IQR)	> 20 *n* = 19 Median (IQR)	H-statistic (*p*-value)		Female, median (IQR)	Male median (IQR)	H statistic (*p*-value)
HAQ-DI (0–3)	0.62 (0.25–1.25)	1.12 (0.6–1.5)	1.37 (0.9–2)	12.686 (0.002)	**0.240 (0.004)**	0.81 (0.25–1.5)	1.12 (0.65–1.5)	1.251 (0.85–1.4)	1.5 (1.2–2.1)	12.417 (0.006)	**0.312 (**< **0.001)**	1.12 (0.75–1.75)	0.75 (0.75–1.75)	0.022 (0.882)
Pain-VAS (0–100)	15 (0–23)	40 (18–60)	50 (30–70)	16.950 (< 0.001)	**0.283 (**< **0.001)**	25 (10–50)	37.5 (12–58)	50 (20–70)	50 (42–77)	14.484 (0.002)	**0.308 (**< **0.001)**	40 (15–60)	25 (10–78)	0.000 (0.995)
Fatigue-VAS (0–100)	30 (0–50)	45 (20–60)	50 (45–85)	16.040 (< 0.001)	**0.266 (0.001)**	50 (20–65)	45 (15–60)	50 (25–60)	60 (45–80)	5.006 (0.171)	0.145 (0.088)	45 (25–70)	55 (10–68)	0.000 (0.997)
Disease severity(0–100)	30 (10–50)	50 (25–65)	50 (43–70)	12.255 (0.002)	**0.238 (0.005)**	42.5 (15–58)	50 (30–60)	50 (35–55)	65 (42–78)	6.251 (0.099)	**0.186 (0.029)**	50 (25–60)	50 (15–70)	− 0.269 (0.791)
Intestinal problems (0–100)	0 (0–10)	10 (0–35)	10 (0–50)	4.478 (0.096)	**0.166 (0.050)**	0.5 (0–35)	10 (0–42)	10 (0–40)	30 (0–58)	5.636 (0.131)	**0.198 (0.020)**	10 (0–50)	0 (0–5)	3.221 (0.073)
Breathing problems (0–100)	0 (0–10)	20 (0–50)	30 (0–60)	16.290 (< 0.001)	**0.222 (0.008)**	10 (0–50)	12.5 (0–30)	25 (15–50)	30 (15–50)	6.203 (0.102)	**0.191 (0.024)**	20 (0–50)	25 (5–65)	0.835 (0.361)
Raynaud’s attacks (0–100)	35 (0–60)	50 (20–72)	50 (10–70)	1.091 (0.580)	0.108 (0.202)	50 (10–70)	32.5 (0–55)	50 (10–75)	60 (20–80)	4.253 (0.235)	0.046 (0.593)	50 (15–70)	50 (0–75)	0.108 (0.743)
Finger ulceration (0–100)	0 (0–25)	0 (0–40)	0 (0–25)	0.386 (0.825)	0.019 (0.827)	0 (0–10)	5.25 (0–38)	0 (0–40)	0 (0–35)	3.801 (0.283)	0.113 (0.184)	0 (0–30)	0 (0–25)	0.115 (0.734)

Bold values indicate the level of significance *p* ≤ 0.05

*r*_*s*_ Spearman’s correlation coefficient

Table 4 presents differences in health problems by educational background. The differences were significant for pain, fatigue, disease severity and Raynaud’s attacks. Patients with higher education had better (lower) scores than those with only primary education or vocational education.

**Table 4:** Differences in health problems by educational background

Studied variables (score range)	Educational background				
	Basic *n* = 16 Median (IQR)	Professional *n* = 32 Median (IQR)	Secondary *n* = 56 Median (IQR)	Higher *n* = 36 Median (IQR)	H-statistic (*p*-value)
HAQ DI (0–3)	1.25 (0.9–1.8)	1.37 (0.6–1.9)	1.06 (0.75–1.7)	0.75 (0.25–1.25)	7.378 (0.061)
Pain-VAS (0–100)	52.5 (35–75)	50 (25–80)	37.5 (20–50)	15 (0–50)	13.577 (**0.004**)
Fatigue-VAS (0–100)	50 (50–90)	52.5 (30–80)	50 (25–73)	37.5 (15–50)	10.852 (**0.013**)
Disease severity (0–100)	55 (50–75)	50 (38–70)	50 (25–60)	32.5 (18–50)	10.352 (**0.016**)
Intestinal problems (0–100)	22.5 (0–70)	10 (0–20)	10 (0–45)	0 (0–50)	2.724 (0.436)
Breathing problems (0–3)	7.75 (0–35)	22.5 (10–55)	27.5 (0–50)	17.5 (0–30)	3.135 (0.371)
Raynaud’s attacks (0–100)	60 (15–70)	57.5 (25–88)	42.5 (20–70)	22.5 (0–50)	11.680 (**0.009**)
Finger ulceration (0–100)	0.5 (0–25)	0 (0–35)	5.25 (0–40)	0 (0–10)	5.048 (0.168)

Bold values indicate the level of significance *p* ≤ 0.05

*IQR* interquartile range, presented as Q25–Q75, *Q25* first quartile (25th percentile), *Q75* third quartile (75th percentile)

## Discussion

The main aim of this study was to determine the relationship between functional disability, health problems and perceived educational needs of people with SSc. We sought to test the hypothesis that increasing health problems would be associated with higher educational needs. Our results have shown that health problems especially, pain, disability, fatigue and disease severity, are associated with patients’ educational needs. The higher the magnitude of the health problems means the greater the need for patient education. This means those patients with higher health problems are most likely to benefit from patient education. Although all patients with a long-term condition will need patient education at some point in their disease trajectory, identifying those with the highest needs may help to prioritise resources and activities.

While the age of patients and duration of their illness was significantly associated with the severity of their health problems, there were no direct correlations between educational needs and age or disease duration. In other rheumatic diseases, educational needs have been shown to be associated with personal characteristics such as age, gender and disease duration [16–18]. In rheumatoid arthritis (RA) for example, lower age and shorter disease duration is associated with higher educational needs [18] and this has also been shown in systemic lupus erythematosus, where younger age and shorter disease duration were each associated with higher level of educational needs particularly the emotional/feelings domain [16]. In SSc, however, such relationship has not been evident [19]. One plausible explanation for this is that in SSc, educational needs may be more related to the health problems than they are to age and disease duration; and as such, targeting the need or health problems may be more important than targeting age groups for example.

Patients in this study had ‘moderate to severe’ functional disability which is consistent with other findings in SSc [20]. Pain and fatigue may be important determinants of functional disability in patients with limited and diffused forms of the disease [21, 22] and these were also high scoring health problems in our study. The highest educational needs were in the domains of disease process, self-help and managing pain. In most rheumatic diseases, education about disease process has consistently been among the highest scoring ENAT domains [16–19], and there may be a strong message to health practitioners to expect and prepare patient education resources about disease process as a minimum standard, especially when planning group educational interventions. Within the context of multidisciplinary team care, patients with SSc that are identified as having severe disability or high levels of fatigue or pain could be referred for patient education as a standard and this will help ensure an appropriate follow-up/care is in place.

This study has three main limitations. First, although the sample was taken from six sites, it was hospital-based and may not be representative of the SSc population in Poland. As SSc is a rare condition, recruiting from multiple sites is necessary and yet it is difficult to ensure representation. Second, since this was a survey, selection bias cannot be excluded as most motivated patients are more likely to return the questionnaires i.e. self-selection bias. Third, as this was a non-randomised cross-sectional study the relationship between variables represent association rather than causality.

## Conclusion

Functional disability, pain, fatigue and other health problems in people with SSc are associated with considerable educational needs, therefore, health professionals should take this into account when planning patient education for this group of patients. While individualised patient education should target patients’ educational needs, group interventions should consider providing patient education related to disease process as a minimum provision. Using tools such as the ENAT for assessing educational needs may help in to accurately identify patients’ needs, problems and preferences, which is necessary for delivering needs-based patient education.
